# Editorial Note: Brain MRI detection and classification: Harnessing convolutional neural networks and multi-level thresholding

**DOI:** 10.1371/journal.pone.0356571

**Published:** 2026-08-20

**Authors:** The PLOS One Editors

The *PLOS One* Editors issue this Editorial Note to inform readers of the following issues that were noted after this article’s [1] publication:

- Works cited in this article [1] as References 12, 19, and 20 were retracted before the article’s publication date. PLOS considers that these references are not crucial in supporting the research or conclusions reported in [1], but the following sentences in the Literature review are no longer supported:
  - The first sentence of the third paragraph: “An augmentation-based 2D convolutional neural network (CNN) system was proposed by Chanu et al.”
  - The second sentence of the fifth paragraph: “Arpit Kumar Sharma et al. [19] designed a technique based on the modified ResNet50 architecture and enhanced watershed (EWS) algorithm to distinguish between pathological and normal brain MR scans.”

In addition, the article’s Data Availability statement is incorrect and is updated to:

The data underlying the results presented in this study are publicly available from the Harvard Whole Brain Atlas (http://www.med.harvard.edu/AANLIB/). The authors did not receive any special access privileges, and all data are accessible to other researchers under the same conditions. The specific Whole Brain Atlas cases used in this study are listed in S1 Table.

## Supporting information

S1 Table Data availability table. The specific Whole Brain Atlas cases used in this study.
(DOCX)
