# Supplementary material for: Editorial Note: Brain MRI detection and classification: Harnessing convolutional neural networks and multi-level thresholding
Source: PLoS One. 2026 Aug 20;21(8):e0356571. doi: 10.1371/journal.pone.0356571 (PMC13492774; doi:10.1371/journal.pone.0356571)
Supplement: Supplementary file 1 [file pone.0356571.s001.docx]

| **Disease Group** | **Study Category** | **Whole Brain Atlas Case Title** | **URL** |
| --- | --- | --- | --- |
| Normal | Normal | Normal aging: structure and function | https://www.med.harvard.edu/AANLIB/cases/case9/mr1/020.html |
| Normal | Normal | Normal aging: structure and function | https://www.med.harvard.edu/AANLIB/cases/case36/mr1/020.html |
| Neoplastic Disease (Brain Tumor) | Glioma | Anaplastic Astrocytoma | https://www.med.harvard.edu/AANLIB/cases/case1/mr1/029.html |
| Neoplastic Disease (Brain Tumor) | Glioma | Astrocytoma | https://www.med.harvard.edu/AANLIB/cases/caseSLU2/mr2/012.html |
| Neoplastic Disease (Brain Tumor) | Glioma | Astrocytoma | https://www.med.harvard.edu/AANLIB/cases/case31/mr1/017.html |
| Neoplastic Disease (Brain Tumor) | Metastatic Bronchogenic Carcinoma | Metastatic Bronchogenic Carcinoma | https://www.med.harvard.edu/AANLIB/cases/case28/mr1-tc1/010.html |
| Neoplastic Disease (Brain Tumor) | Metastatic Adenocarcinoma | Metastatic Carcinoma of the Colon | https://www.med.harvard.edu/AANLIB/cases/case26/mr1/016.html |
| Neoplastic Disease (Brain Tumor) | Meningioma | Meningioma | https://www.med.harvard.edu/AANLIB/cases/case32/mr4/017.html |
| Neoplastic Disease (Brain Tumor) | Sarcoma | Sarcoma | https://www.med.harvard.edu/AANLIB/cases/case33/mr1/017.html |
| Degenerative Disease | Alzheimer's Disease | Alzheimer's Disease | https://www.med.harvard.edu/AANLIB/cases/case3/mr1/020.html |
| Degenerative Disease | Alzheimer's Disease | Alzheimer's Disease | https://www.med.harvard.edu/AANLIB/cases/case29/mr1/017.html |
| Degenerative Disease | Huntington's Disease | Huntington's Disease | https://www.med.harvard.edu/AANLIB/cases/case11/mr1/011.html |
| Inflammatory or Infectious Disease | Multiple Sclerosis | Multiple Sclerosis | https://www.med.harvard.edu/AANLIB/cases/case5/mr10/020.html |
| Inflammatory or Infectious Disease | Multiple Sclerosis | Multiple Sclerosis | https://www.med.harvard.edu/AANLIB/cases/case38/mr1/013.html |
